# Evaluating the Interaction of Emerging Diseases on White-Tailed Deer Populations Using an Agent-Based Modeling Approach

**DOI:** 10.3390/pathogens13070545

**Published:** 2024-06-28

**Authors:** Miranda Strasburg, Sonja Christensen

**Affiliations:** Department of Fisheries and Wildlife, Michigan State University, East Lansing, MI 48824, USA; mlstrasb@gmail.com

**Keywords:** chronic wasting disease, hemorrhagic disease, white-tailed deer, population impacts

## Abstract

Disease co-occurrence in wildlife populations is common yet understudied. In the case of disease-caused mortality, the mortality attributed to one disease has the potential to buffer populations against subsequent alternative disease outbreaks by reducing populations and thus contacts needed to sustain disease transmission. However, substantial disease-driven population declines may also prevent populations from recovering, leading to localized extinctions. Hemorrhagic disease (HD), a vector-transmitted, viral disease in white-tailed deer (WTD), similar to chronic wasting disease (CWD), a prion disease, has increased in frequency and distribution in the United States. However, unlike CWD, which progresses slowly, HD can cause mortality only days after infection. Hemorrhagic disease outbreaks can result in substantial localized mortality events in WTD near vector habitats such as wetlands and may reduce local deer densities and consequent CWD transmission. The objective of our study was to evaluate the potential for HD outbreaks to buffer CWD risk where the diseases co-occur. Using an agent-based modeling approach, we found that frequent, intense HD outbreaks have the potential to mitigate CWD risk, especially if those outbreaks occur shortly after CWD introduction. However, HD outbreaks that do not result in substantial WTD mortality are unlikely to impact CWD or WTD population dynamics. Severe HD outbreaks may reduce CWD cases and could present an opportunity for managers to boost CWD control initiatives in a post-HD outbreak year.

## 1. Introduction

Wildlife populations are commonly infected with multiple pathogens, and disease coinfection is likely the norm among populations rather than the exception [1,2,3]. However, at the wildlife population scale, the occurrence of one disease in a population has the potential to buffer populations against subsequent disease outbreaks if the initial disease functionally reduces contact rates and thus transmission among animals. By reducing populations below the threshold needed to sustain disease transmission between infected and susceptible individuals, density-dependent disease transmission will slow. Nonetheless, substantial population declines caused by disease may prevent populations from recovering, leading to localized extinctions (reviewed in [4]). With wildlife diseases emerging at unprecedented rates [5,6], investigating potential interactions between diseases is vital for wildlife conservation.

White-tailed deer (WTD) populations are increasingly threatened by the introduction and spread of novel diseases. Chronic wasting disease (CWD), a prion disease that infects members of the cervid family, has expanded across the United States (US) since its discovery in the 1960s in Colorado [7]. Likewise, hemorrhagic disease (HD), a vector-transmitted disease caused by the orbiviruses epizootic hemorrhagic disease virus or blue tongue virus, has increased in frequency and distribution in WTD across northern latitudes since the 1980s [8]. Both HD and CWD can cause mortality in WTD, but they act at different temporal scales. CWD progresses very slowly, generally leading to death about two years after infection [9], whereas HD can cause mortality only days after infection [10]. In contrast to CWD, which persists in the environment [11], HD outbreaks are cyclic, occurring every five to seven years, and tend to be associated with warm summer temperatures [12] and reduced precipitation [13]. HD outbreaks can result in substantial localized mortality events in WTD, particularly near wetland and riverine habitats [14,15], which are critical for reproduction in the vector species, biting midges in the *Culicoides* genus, responsible for transmitting HD [16]. Factors that reduce local WTD density, like HD, have the potential to mitigate CWD risk by reducing the probability of contact between susceptible and infected deer through reductions in deer density. Understanding how HD and CWD may interact to influence WTD populations is critical given the cultural, economic, and ecological importance of WTD [17,18].

The objective of our study was to determine how the intensity, frequency, and timing of HD outbreaks affect CWD and WTD population dynamics. We hypothesized that HD outbreaks would alter CWD risk (i.e., total cases, prevalence, and persistence). We predicted that increases in HD outbreak frequency and intensity would reduce CWD risk, leading to reductions in CWD cases, prevalence, and persistence. We also predicted that HD outbreaks that occurred shortly after CWD was introduced would have a higher potential to buffer CWD by directly reducing the number of CWD-infected individuals than HD outbreaks that occurred before CWD was introduced. We tested our hypothesis using a set of previously published agent-based models (ABMs) that were created to simulate CWD dynamics in WTD populations in Michigan counties [19,20].

## 2. Materials and Methods

### 2.1. Agent-Based Model Framework

The OvCWD agent-based modeling framework is composed of two separate ABMs, OvPop (*Odocoileus virginianus* Population model version 1.1.0) and OvCWDdy (*O. virginianus* CWD dynamics model version 1.0.0), coded in the NetLogo modeling environment, and it was developed to connect WTD demography and behavior to dynamics of CWD [19,20]. OvPop simulates population dynamics using population and behavior parameters (Supplemental Table S1) and mortality rates (Supplemental Table S2) at a one-month timestep for 25 years, allowing the population growth rate to stabilize at lambda equal to 1.0 and reach equilibrium. The resulting population snapshot with details on sex, age, and group composition is then used in the OvCWDdy model to simulate CWD entering a population with a stable growth rate and age–sex structure. We determined the initial deer populations in our model using the same model parameters used to model CWD dynamics in WTD in Michigan [21]. The OvCWDdy model simulates disease and population dynamics for 10 years following CWD introduction. Source code and detailed ODD protocols (Overview, Design concepts, and Details) are available for the OvPop and OvCWDdy models [19,20], but we will describe model components that are particularly relevant to this study.

During each timestep in the OvCWDdy model, contacts between infected and susceptible deer are modeled using a user-specified deer contact network. This contact network specifies the minimum and maximum number of contacts individual deer are expected to have with other deer based on the published literature (see [21] for additional contact network description). When deer interact in the model, a random number between the minimum and maximum number of contacts is selected, and each of those contacts has a disease transmission rate of 0.0128. The number of contacts between deer varies depending on each deer’s age, sex, and group membership (i.e., doe social groups, male bachelor groups). For instance, in the model, fawns 3 months of age have 60–90 contacts with their mothers per month, but as they age, the minimum and maximum number of contacts decreases (e.g., 4–6 months: 20–30 contacts, 7–9 months: 5–10 contacts). The model also simulates doe and bachelor group behaviors, with deer in the same group having more contacts and therefore higher probabilities of CWD transmission (e.g., solitary males have between 1 and 5 contacts per month with other solitary males, whereas males in bachelor groups have between 5 and 15 contacts with other group members).

There are no treatments available for CWD, so the only current method to control CWD spread in white-tailed deer is to limit contact between individuals by removing deer from the landscape. The modeling original framework has four ways that CWD-positive deer can be removed: (1) emigration, (2) human harvest (i.e., hunting), (3) non-specified natural mortality, and (4) mortality due to CWD. The landscape in the modeling framework is a torus, which means if a deer disperses beyond the bounds of the landscape, the deer are “wrapped” so that they reappear on the opposite edge. If a dispersing (emigrating) deer is CWD-positive, it will wrap and reappear (immigrate) into the model as a CWD-negative deer. Deer can also be removed by human harvest and natural mortality, and sex- and age-specific rates can be specified by users in the NetLogo interface (Supplemental Table S2). Finally, CWD-positive deer die when their disease progression has advanced to clinical stages. In the model environment, a CWD-positive deer begins transmitting the disease via contacts 6–10 months post-infection and remains in this infectious stage for an initial 11–15 months at which time it enters the clinical stage of the disease. CWD-driven mortality occurs 1–2 months after the deer reaches clinical stage. Deer in the clinical disease stage continue to transmit CWD via contacts but do not participate in mating. In this framework modeled here, deer are also removed from the model environment due to HD outbreaks. 

The original OvCWDdy modeling framework was intended to simulate CWD dynamics at the county level, and the underlying model landscape was determined based on the percent forest canopy cover at a 1-mile spatial resolution, as it was designed to match the measurement units most used by management agencies and evidence on deer habitat metrics of significance [22]. Within the original model environment, landscape pixels with percent forest cover between 25% and 75% were considered “deer habitat”. Depending on the forest cover of neighboring pixels, pixels that did not meet this initial threshold were combined and considered “deer habitat”. Ultimately, this created heterogeneity in deer density based on the underlying landscape’s forest cover, such that habitat patches that were not optimal received fewer deer than optimal habitat. In this analysis, these deer habitat pixels were initially seeded with a deer density of 10 deer per square mile. In addition to the initial population seeding, forest cover also influences juvenile male dispersal behavior in the modeling framework, such that males disperse farther in areas with less forest cover [23]. Yearling male dispersal distance is described by a linear relationship where the dispersal distance is zero in areas where forest cover exceeds 72% [23]. Female deer were also dispersed in the models, but those movements were not influenced by landscape composition.

We used the ‘nlm_randomcluster’ function in the ‘NLMR’ package in R to simulate random neutral landscape models composed equally of two habitat types, “deer habitat” and “non-habitat” [24]. Neutral landscape models use theoretical distributions to simulate landscape patterns [25]. To simulate fragmented habitats, which realistically represent landscapes commonly used by WTD, we set the clustering parameter to 0.1, such that only 10% of the “deer habitat” in each landscape was clustered together. The selection of the clustering parameter was based on Rahimi et al. [26], who used the ‘nlm_randomcluster’ function to generate landscapes with high and low levels of fragmentation. We reclassified the landscapes generated by the ‘nlm_randomcluster’ analysis (originally binary rasters with 1 representing deer habitat and 0 representing non-habitat) so that the maximum value in the raster was 0.50, or 50% forest cover, so that deer habitat was represented within the NetLogo modeling environment. We then smoothed the resulting landscape to better mimic natural transitions between deer habitat and non-habitat and heterogeneity in deer density. We created 576 mi^2^ (24 by 24 miles) landscapes to represent an area similar to many Midwestern counties (the median county area for Michigan is 567 mi^2^). We repeated this process to create 10 replicate landscapes.

### 2.2. Experimental Design

**Figure 1:**
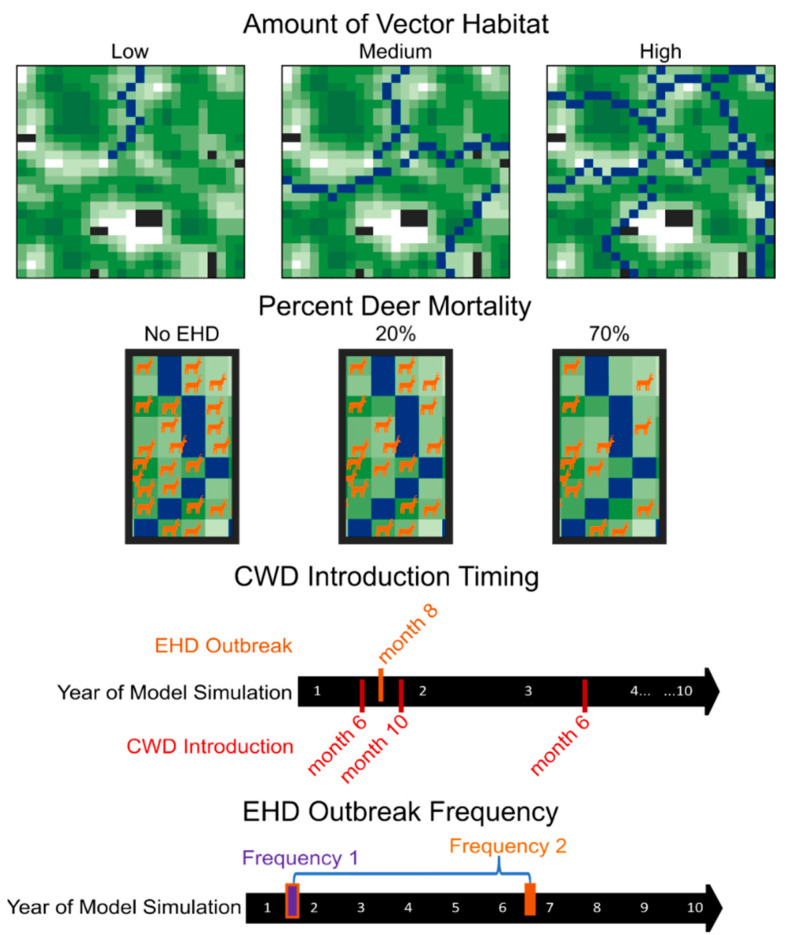
Schematic diagram of experimental design. We modeled chronic wasting disease dynamics across 36 different HD treatment combinations (3 levels of vector habitat abundance [low, medium, or high] × 2 levels of percent deer mortality [20% or 70%]) × 3 HD outbreak timing treatments [2 months before CWD introduction, 2 months after CWD introduction, or 26 months after CWD introduction] × 2 HD outbreak frequencies [1 outbreak per simulation, 2 outbreaks per simulation = 36 HD treatments] and 2 disease control scenarios (HD-absent control or CWD-absent control) for 10 replicate landscapes. The landscapes used in the modeling environment were derived from neutral landscape models and thus represent virtual space. In each landscape, black areas represent areas that are not occupied by deer, and shades of green represent relative percent forest cover with darker areas indicating higher cover. Blue areas represent vector (i.e., river) habitats.

We manipulated HD outbreak intensity (amount of vector habitat [low, medium, or high] and percent deer mortality [20% or 70%]), HD outbreak timing (2 months before CWD introduction, 2 months after CWD introduction, or 26 months after CWD introduction), and HD outbreak frequency (1 outbreak per simulation and 2 outbreaks per simulation), resulting in 36 treatments total (3 vector habitat treatments × 2 percent morality treatments × 3 HD timing treatments × 2 HD frequencies = 36 treatments; Figure 1). We replicated each treatment on 10 distinct landscapes for 100 model iterations. For each landscape, we also ran models without CWD introduction and with CWD but without HD outbreaks to represent our disease control scenarios, which ultimately created 38 total treatment groups.

### 2.3. Simulating HD Dynamics

The vector species associated with HD rely on wet, muddy soils for reproduction [16] and thus, HD outbreaks are commonly associated with wetland or riparian habitats [14,15]. This leads to localized mortality effects near wetlands or river sites, generally in the late summer or early fall. With this in mind, we randomly categorized pixels within our landscapes as river patches, but more generally speaking, these river patches can be viewed as areas on landscapes with high risk for HD outbreaks. In models with an HD outbreak, we simulated HD-driven mortality events by removing deer agents from areas within a 1-mile radius of a river patch, with the number of deer removed from the landscape depending on the abundance of river patches. We created three levels of vector habitat abundance (i.e., low, medium, and high) by changing the number of rivers present in the model landscape. Rivers were randomly created from the center of the model landscape to its edge. Landscapes with low, medium, and high vector habitat abundance had one, four, or eight rivers, respectively. Ultimately, this led to identifying, on average, 2, 8, and 16% of the landscape for low, medium, and high vector habitat abundance as river habitats. We also changed the severity of each HD outbreak by altering the number of deer that were removed from the landscape during an HD outbreak event. We simulated low- and high-severity outbreaks by removing 20% and 70%, respectively, of deer near the river patches.

We also manipulated the timing and frequency in which outbreaks of HD occurred. We changed the timing of the first HD outbreak occurrence in relation to CWD introduction. We modeled three different HD timing scenarios. Under our first scenario, the initial HD outbreak occurred 2 months before CWD was introduced onto the landscape, ultimately reducing the initial deer abundance on the landscape. Under our second scenario, the initial HD outbreak occurred 2 months after CWD was introduced on the landscape, thereby reducing the deer population before substantial CWD spread, and increasing the likelihood of removing an initial CWD-positive animal. In our final scenario, the initial HD outbreak occurred 26 months after CWD introduction, allowing for CWD spread prior to a population reduction. We included these timing scenarios to mimic the two main modes in which CWD is likely to get introduced into a new area, the first being in early spring when juvenile deer, typically males, disperse from their family group into new territories, and the second during hunting seasons when deer carcasses are transported from one area to another. CWD was only introduced once, and it was introduced by a young male. Because HD outbreaks are cyclic, occurring once every five to ten years, we modeled the frequency of outbreak occurrence once or twice within the 10-year simulation. In the scenarios where two HD outbreaks occurred, a second outbreak occurred five years after the initial outbreak. Given that HD outbreaks typically occur during the late summer or early fall, all HD outbreaks occurred during August (month 8) of our model simulations.

Deer populations that experience an HD outbreak have been shown to bounce back after HD outbreak events [15], suggesting that density-dependent population growth may be at play in some WTD populations. To simulate this in the ABM environment, we increased the pregnancy rate of does within one mile of a river pixel by 20% after an HD outbreak occurred. The pregnancy rate remained elevated until the population was within 1% of the initial population size at the beginning of the simulation, at which time the pregnancy rate was reduced to the original rates used to establish a stable population. This ultimately simulated the reduction in competition between individuals after an HD outbreak and consequent increased fitness and reproductive capacity, as we would expect to see in wild populations [27].

### 2.4. Response Variables and Statistical Analysis

The OvCWDdy model outputs the total deer population, the number of CWD-positive deer, and the number of CWD-positive landscape pixels (i.e., total CWD area). Only landscape pixels with active CWD cases at the end of the simulation were used to calculate the total CWD area. The ABM used in this study does not have a mechanism to simulate environment transmission, so infected deer do not contaminate landscape pixels as they move through the environment. We used these outputs to calculate CWD prevalence and the percent of total outcomes across all scenarios when an outbreak of CWD occurred. CWD prevalence is defined as the total number of CWD-positive deer in the population divided by the total population. The percent of outcomes in a CWD outbreak that occurred represents the proportion of model iterations in which CWD persisted for the entire simulation and ended with a prevalence rate of ≥1%. We used a generalized linear mixed effects model (GLMM) with a binomial error distribution to test for treatment effects on the percent of times CWD introduction led to an outbreak with the landscape replicate as a random factor. The mean differences between the number of CWD-positive deer, landscape pixels, and CWD prevalence given that CWD persisted on the landscape among HD treatments were determined by a one-way analysis of variance (ANOVA), followed by a Dunnett’s test to compare HD treatments and no-HD control treatment with the responses average by replicate landscape. We also used a one-way ANOVA, followed by a Dunnett’s test, to determine the mean differences in total deer population size among treatments and the no-CWD control for each landscape replicate. All analyses were performed in R version 4.1.2.

## 3. Results

**Figure 2:**
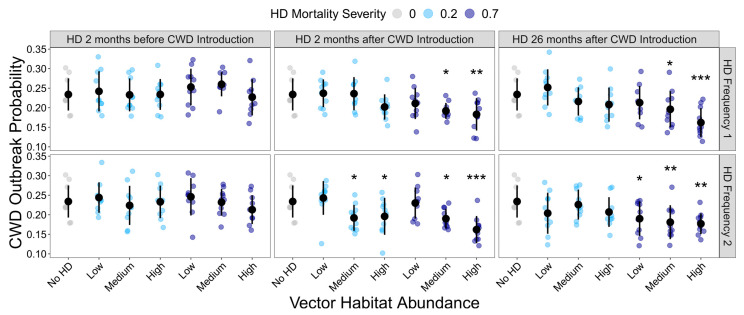
Influence of HD treatment on the CWD outbreak probability. The proportion of model simulations that resulted in CWD prevalence ≥ 1% at the end of the 10-year model simulations for each HD treatment. HD treatments differed in vector habitat abundance (low, medium, or high), mortality severity (20% or 70%), frequency (1 or 2 per 10-year simulation), and timing (2 months before CWD introduction, 2 months after CWD introduction, or 26 months after CWD introduction). Plotted values are means ± 1 SD. The blue and gray points indicate the average CWD outbreak probability for each of the ten replicate landscapes. * *p* < 0.05, ** *p* < 0.01, *** *p* < 0.001.

HD treatment significantly influenced the probability of the occurrence of a CWD outbreak (GLMM: χ² = 141.01, df = 36, *p* < 0.001). However, not all HD treatments differed significantly from the HD-absent control (Figure 2; Supplemental Table S3). Generally, as the frequency and severity of HD outbreaks increased, the probability of CWD outbreak occurrence declined. However, the initial timing of the HD outbreak greatly influenced the buffering capacity of HD on CWD risk. HD outbreaks, regardless of their intensity or frequency, had no impact on CWD persistence if they initially occurred prior to CWD being introduced.

**Figure 3:**
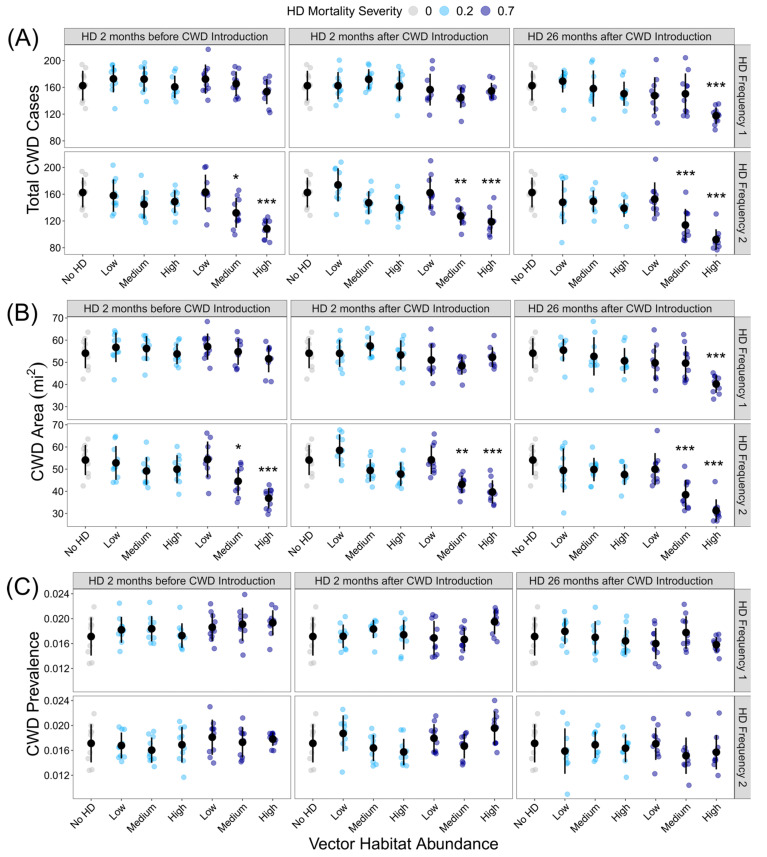
Influence of HD treatment on the CWD risk: (**A**) total CWD cases at the end of the 10-year model simulations for each HD treatment; (**B**) CWD area at the end of the 10-year model simulation for each HD treatment; (**C**) CWD prevalence at the end of the 10-year model simulation. HD treatments differed in vector habitat abundance (low, medium, or high), mortality severity (20% or 70%), frequency (1 or 2 per 10-year simulation), and timing (2 months before CWD introduction, 2 months after CWD introduction, or 26 months after CWD introduction). Plotted values are means ± 1 SD. The blue and gray points indicate the average variable for each of the ten replicate landscapes. * *p* < 0.05, ** *p* < 0.01, *** *p* < 0.001.

HD treatment significantly influenced the total CWD cases (F_36,333_= 8.849, *p* < 0.001) and total CWD area (F_36,333_= 9.809, *p* < 0.001), but as with outbreak probability, not all HD treatments were significantly different from the HD-absent control (Figure 3A,B; Supplemental Tables S4 and S5). If intense and frequent HD outbreaks occurred during the model simulation, the total number of CWD cases and CWD area was reduced, regardless of the timing of the HD outbreak. HD treatment significantly influenced CWD prevalence (F_36,333_= 2.374, *p* < 0.001), but post hoc analyses revealed that no HD treatments were significantly different from the HD-absent control (Figure 3C; Supplemental Table S6).

**Figure 4:**
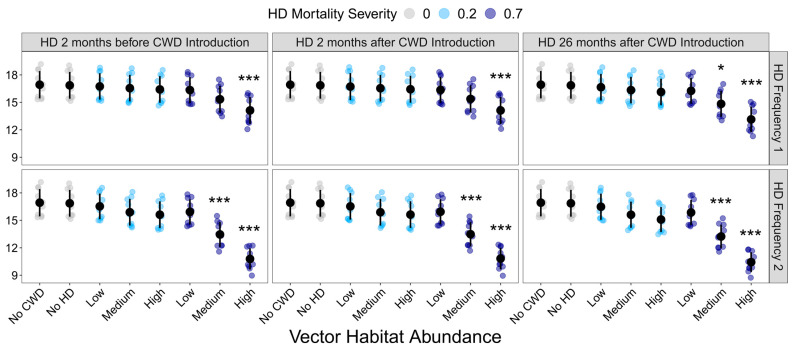
Influence of HD treatment on the final deer population density. Final deer population at the end of the 10-year model simulations for each HD treatment. HD treatments differed in vector habitat abundance (low, medium, or high), mortality severity (20% or 70%), frequency (1 or 2 per 10-year simulation), and timing (2 months before CWD introduction, 2 months after CWD introduction, or 26 months after CWD introduction). Plotted values are means ± 1 SD. The blue and gray points indicate the average deer density for each of the ten replicate landscapes. * *p* < 0.05, *** *p* < 0.001.

Final deer population density was also significantly influenced by disease treatment (F_37,342_= 15.094, *p* < 0.001). Compared to the CWD-absent control, CWD presence alone had no impact on final deer population density (Figure 4; Supplemental Table S7). Similarly, HD treatment only significantly influenced final population density when the resulting mortality was extreme. HD had no effect on population density in landscapes with low vector habitat abundance or 20% deer mortality (Figure 4).

## 4. Discussion

Although wildlife populations are commonly plagued by multiple diseases, how diseases interact to influence populations is rarely investigated [3,28]. When disease coinfection experiments are carried out, they are often limited to diseases with similar modes of transmission [29], causal agents [30,31], or infection model organisms that are easy to manipulate in the laboratory [32]. Executing small-scale experiments with large, economically important species like WTD is costly, and designing broad-scale experiments with sufficient replication is impractical. Working with complex, unpredictable diseases, like HD and CWD, only adds to the challenge. Fortunately, disease models, like the agent-based modeling approach used in this study, allow for the rapid simulation of these two complicated diseases to address questions that would be impossible to answer using real-world experiments. Here, we show that HD outbreaks do have the capacity to buffer CWD risk within WTD populations, but this potential is only achieved when HD outbreaks result in substantial mortality and occur frequently. Likewise, the effect of HD outbreaks on CWD risk is reduced if the HD outbreak occurs prior to CWD establishment. As environmental conditions continue to shift favoring HD vectors, and CWD cases continue to rise, we can anticipate an increased potential for the overlap of CWD and HD; this study helps us begin to understand how interactions between these diseases could influence WTD populations.

Reductions in deer density have the potential to reduce CWD transmission between infected and uninfected deer. In fact, reducing deer densities through extensive culling or increased hunter harvest is one of the most used strategies to mitigate CWD [33,34]. We were motivated to perform this study because HD outbreaks in natural and captive populations can result in substantial, localized mortality [15,35,36,37] and as such may naturally buffer populations against CWD. However, the frequency and intensity of HD is highly variable [31,38], and the impact of HD on deer populations depends heavily on whether the disease occurs in enzootic or epizootic areas [39]. In naïve populations with no immunity or disease resistance, HD causes mortality [39] and can drastically reduce deer densities, particularly near river corridors [15]. However, in endemic areas, though HD seroprevalence is high, clinical symptoms and mortality are not common [40]. We modeled these differences by changing the number of deer that were removed from the landscape during an HD event, both by changing the extent of the outbreak (i.e., amount of vector habitat) and the percent of deer that were removed during an outbreak from that habitat. Our results suggest that in endemic areas where mortality is low (≤20%), HD is unlikely to reduce the CWD risk. However, in emerging disease zones, there is potential for HD outbreaks to mitigate CWD in populations, as is evident by our 70% deer mortality scenario. This high-mortality scenario is not impossible, as Christensen et al. [15] found an over 75% recovery in deer density from one year after the EHD outbreak to five years post-outbreak near a river corridor in central Michigan. Although the pre-HD outbreak density was unknown for the area, population increases following the outbreak year were absent both in agricultural areas adjacent to the river and in a comparable riverine habitat where no mortalities were observed [15], suggesting that HD was the cause of the local decline. The 75% recovery (or increase) in deer density 5 years following an HD outbreak suggests that the HD outbreak caused a large reduction in deer density (>70%) that rebounded over time, which supports that a mortality rate of 70% as we modeled in our high-deer-mortality scenario could happen in natural environments.

Our results suggest that the amount of vector habitat in a landscape directly impacts the probability of HD outbreaks affecting reduced CWD risk. Unsurprisingly, we found that increases in vector habitat reduced CWD risk following an HD outbreak. As more deer are removed from the landscape during an HD mortality event, there is a higher probability of randomly removing a deer with CWD and reducing subsequent transmission events. Although it is difficult to predict future HD impacts given its dependence on a variety of factors including the abundance of wetland habitat [14], temperature [12], and precipitation [13] in addition to the potential for deer populations to develop immunity against viruses that cause HD [39], this study highlights that under certain conditions, HD outbreak can impact CWD risk in WTD populations.

The capacity for HD to influence CWD depends heavily on the timing of the HD outbreak. The fast-acting and localized disease impacts from HD present a stark contrast in disease dynamics from CWD, which generally progresses slowly both in individual animals and at the population level. We found that HD outbreaks do not reduce the probability of a CWD outbreak if they occur prior to CWD introduction despite reducing the density of deer under high-severity and -frequency scenarios, at least in our simulations. If the HD outbreaks occurred after CWD was introduced, in those same high-severity and -frequency scenarios, HD did reduce CWD outbreak potential. Ultimately, the possibility of removing an initial case of CWD within a deer population and in an area with otherwise very low CWD prevalence has significant effects on the likelihood of CWD outbreak and persistence. This is because CWD transmission is not entirely density-dependent, and instead the frequency of infected individuals within a population can influence CWD dynamics [41,42]. If density-dependent mechanisms were solely at play with CWD, reducing the total number of deer, and thus their density, would always reduce CWD risk. We did not observe that in our model; HD outbreaks had the greatest effect when CWD was introduced just prior to the HD outbreak. HD outbreaks only have the capacity to reduce the number of infected individuals and thus the frequency of CWD, if CWD is already present within the population. Our modeling approach allows us to know exactly when CWD is introduced into the population, but on natural landscapes, CWD can go undetected in a population for years due to its slow progression, so knowing when CWD is introduced into a population is nearly impossible. Again, as HD outbreak frequency increases, the likelihood of occurring early after CWD increases, and therefore, frequent HD outbreaks are the most likely to impact CWD dynamics.

CWD risk is often associated with reported disease prevalence, which is the number of infected individuals out of the total number of individuals tested. Modeling techniques, like the one used in this study, allow the user to quantify true prevalence, a number that is impossible to obtain from free-ranging cervid populations. In this study, we found that HD outbreaks have no impact on true CWD prevalence, and that is because, while severe HD outbreaks may reduce the number of CWD-positive individuals, they also reduce the total number of deer on the landscape. This ultimately leads to no changes in true prevalence but rather a reduction in the absolute value of both infected and non-infected deer. In other words, there is no bias in the reduction in CWD-infected vs. non-infected individuals. This highlights that managers must consider the total population size when determining CWD surveillance strategies because disregarding how many deer occur on the landscape when deciding how many deer to test for CWD may prevent CWD detection [43].

Our study fails to incorporate behavioral changes associated with CWD infection in deer that likely make CWD-positive deer more likely to contract HD. A key symptom of CWD is excessive thirst and increased drinking (Williams et al., 2002). This symptom may draw CWD-positive deer toward water sources, where they also may be more likely to contract HD via contact with HD vectors. In our model, CWD infection did not alter water-seeking behavior in deer, and therefore CWD-positive deer would be no more likely to succumb to HD than CWD-negative deer. While no experimental studies to our knowledge have ever challenged CWD-positive deer with a second infection, it is likely that CWD-positive deer would be more vulnerable to subsequent infections due to behavioral or immune responses and therefore may succumb to CWD or HD more quickly, which could result in reduced transmission of CWD to healthy deer. However, in our model, CWD-infected and non-infected deer were equally vulnerable to HD-driven mortality. Increased water-seeking behavior and decreased capacity to fight subsequent infections in CWD-positive deer would increase HD-related impacts on CWD, and as such, our results may underestimate the potential for HD to buffer CWD.

Ultimately, this work highlights that while severe HD outbreaks can potentially reduce localized CWD risk, they are unlikely to completely eradicate CWD from the landscape. Additionally, more realistic, moderate HD-driven mortality events are expected to have little impact on CWD or WTD population dynamics. This suggests that managers should not assume fewer CWD-positive animals following an HD event but instead may consider increasing efforts for deer density management and CWD control strategies following HD outbreaks as these natural reductions in deer densities may provide a unique opportunity to get a leg up on CWD.

## Data Availability

Data can be made available upon request to the authors.

## Supplementary Materials

The following supporting information can be downloaded at: https://www.mdpi.com/article/10.3390/pathogens13070545/s1. Table S1: Population and behavior parameters used in the OvCWDdy modeling environment. Table S2: Age- and sex-specific mortality parameters used in the OvCWDdy modeling environment. Table S3: Generalized linear mixed model output for the impact of HD treatment on the probability of a CWD outbreak. Table S4: Results of Dunnett’s post-hoc analysis for total CWD infected areas. Table S5: Results of Dunnett’s post-hoc analysis for total CWD positive deer. Table S6: Results of Dunnett’s post-hoc analysis for CWD prevalence. Table S7: Results of Dunnett’s post-hoc analysis for deer population density.
