# Supplementary material for: Evaluating the Interaction of Emerging Diseases on White-Tailed Deer Populations Using an Agent-Based Modeling Approach
Source: Pathogens. 2024 Jun 28;13(7):545. doi: 10.3390/pathogens13070545 (PMC11279658; doi:10.3390/pathogens13070545)
Supplement: Supplementary file 1 [file pathogens-13-00545-s001.zip › pathogens-3022963-supplementary.pdf]

pathogens MDPI

Supplemental Tables

Table S1: Population and behavior parameters used in the `OvCWDdy` modeling environment. These values were used in the original `MIOvPop` model (Belsare and Stewart, 2020).

Parameter	Value
**Population Parameters**	
Sex ratio (Male:Female ratio)	1:1.2
Proportion of adults in population	0.4
Proportion of yearlings in the population	0.25
**Behavior Parameters**	
Bachelor groups size	4 ± 1 (mean ± SD)
Doe family group size	6
Yearling male dispersal rate	0.46
Yearling female dispersal rate	0.22
Female dispersal distance	11 ± 4 miles (mean ± SD)

Table S2: Age- and sex-specific mortality parameters used in the `OvCWDdy` modeling environment. These values were used in the original `MIOvPop` and `MIOvCWDdy` models (Belsare and Stewart, 2020).

Parameter	Value
**Non-hunting morality**	
Male and Female Fawns (0-6 months)	0.055 per month
Male and Female Fawns (7-12 months)	0.05 per month
Male Yearlings (13- 24 months)	0.01 per month
Female Yearling (13- 24 months)	0.00 per month
Male Adults (>25 months)	0.01 per month
Female Adults (>25 months)	0.02 per month
**Hunting morality**	
Male and Female Fawns (0-6 months)	0
Male Fawns (7-12 months)	0.07 per year
Female Fawns (7-12 months)	0.09 per year
Male Yearlings (13- 24 months)	0.42 per year
Female Yearling (13- 24 months)	0.22 per year
Male Adults (>25 months)	0.35 per year
Female Adults (>25 months)	0.118 per year

Table S3: Generalized linear mixed model output for the impact of HD treatment on the probability of a CWD outbreak. Significant differences from HD-control are indicated in bold.

Source of Variation	Estimate	SE	*z*	*P*
Intercept	-1.186	0.078	-15.305	<0.001
**HD Timing: 2 months before CWD Introduction**				
HD Severity: 20% Mortality				
HD Severity: Low Vector Habitat				
HD Frequency: 1 per 10-year period	0.044	0.107	0.415	0.678
HD Severity: Low Vector Habitat				
HD Frequency: 2 per 10-year period	0.055	0.106	0.517	0.605
HD Severity: Medium Vector Habitat				
HD Frequency: 1 per 10-year period	-0.006	0.107	-0.052	0.959
HD Severity: Medium Vector Habitat				
HD Frequency: 2 per 10-year period	-0.057	0.108	-0.524	0.600
HD Severity: High Vector Habitat				
HD Frequency: 1 per 10-year period	0.000	0.107	0.001	0.999
HD Severity: High Vector Habitat				
HD Frequency: 2 per 10-year period	-0.006	0.107	-0.051	0.959
HD Severity: 70% Mortality				
HD Severity: Low Vector Habitat				
HD Frequency: 1 per 10-year period	0.103	0.106	0.975	0.330
HD Severity: Low Vector Habitat				
HD Frequency: 2 per 10-year period	0.066	0.106	0.620	0.536
HD Severity: Medium Vector Habitat				
HD Frequency: 1 per 10-year period	0.140	0.106	1.327	0.185
HD Severity: Medium Vector Habitat				
HD Frequency: 2 per 10-year period	-0.011	0.108	-0.104	0.917
HD Severity: High Vector Habitat				
HD Frequency: 1 per 10-year period	-0.039	0.108	-0.366	0.714
HD Severity: High Vector Habitat				
HD Frequency: 2 per 10-year period	-0.121	0.109	-1.112	0.266
**HD Timing: 2 months after CWD Introduction**				
HD Severity: 20% Mortality				
HD Severity: Low Vector Habitat				
HD Frequency: 1 per 10-year period	0.017	0.107	0.156	0.876
HD Severity: Low Vector Habitat				
HD Frequency: 2 per 10-year period	0.050	0.107	0.466	0.641
HD Severity: Medium Vector Habitat				
HD Frequency: 1 per 10-year period	0.011	0.107	0.105	0.917
HD Severity: Medium Vector Habitat				
HD Frequency: 2 per 10-year period	-0.251	0.111	-2.259	0.024
HD Severity: High Vector Habitat	-0.188	0.110	-1.703	0.089

HD Frequency: 1 per 10-year period				
HD Severity: High Vector Habitat HD Frequency: 2 per 10-year period	-0.226	0.111	-2.036	0.042
HD Severity: 70% Mortality				
HD Severity: Low Vector Habitat HD Frequency: 1 per 10-year period	-0.133	0.109	-1.217	0.224
HD Severity: Low Vector Habitat HD Frequency: 2 per 10-year period	-0.022	0.107	-0.208	0.835
HD Severity: Medium Vector Habitat HD Frequency: 1 per 10-year period	-0.251	0.111	-2.258	0.024
HD Severity: Medium Vector Habitat HD Frequency: 2 per 10-year period	-0.264	0.111	-2.371	0.018
HD Severity: High Vector Habitat HD Frequency: 1 per 10-year period	-0.310	0.113	-2.756	0.006
HD Severity: High Vector Habitat HD Frequency: 2 per 10-year period	-0.458	0.115	-3.965	<0.001
**HD Timing: 26 months after CWD Introduction**				
HD Severity: 20% Mortality				
HD Severity: Low Vector Habitat HD Frequency: 1 per 10-year period	0.098	0.106	0.924	0.355
HD Severity: Low Vector Habitat HD Frequency: 2 per 10-year period	-0.176	0.110	-1.600	0.109
HD Severity: Medium Vector Habitat HD Frequency: 1 per 10-year period	-0.103	0.109	-0.950	0.342
HD Severity: Medium Vector Habitat HD Frequency: 2 per 10-year period	-0.045	0.108	-0.418	0.676
HD Severity: High Vector Habitat HD Frequency: 1 per 10-year period	-0.151	0.109	-1.381	0.167
HD Severity: High Vector Habitat HD Frequency: 2 per 10-year period	-0.157	0.110	-1.435	0.151
HD Severity: 70% Mortality				
HD Severity: Low Vector Habitat HD Frequency: 1 per 10-year period	-0.121	0.109	-1.110	0.267
HD Severity: Low Vector Habitat HD Frequency: 2 per 10-year period	-0.264	0.111	-2.369	0.018
HD Severity: Medium Vector Habitat HD Frequency: 1 per 10-year period	-0.226	0.111	-2.037	0.042
HD Severity: Medium Vector Habitat HD Frequency: 2 per 10-year period	-0.324	0.113	-2.877	0.004
HD Severity: High Vector Habitat HD Frequency: 1 per 10-year period	-0.458	0.115	-3.974	<0.001
HD Severity: High Vector Habitat	-0.351	0.113	-3.103	0.002

HD Frequency: 2 per 10-year period

Table S4: Results of Dunnett's post-hoc analysis for total CWD infected areas. Significant differences from HD-control are indicated in bold.

Source of Variation	Difference in Means	Lower CI	Upper CI	*P*
**HD Timing: 2 months before CWD Introduction**				
HD Severity: 20% Mortality				
HD Severity: Low Vector Habitat<br>HD Frequency: 1 per 10-year period	2.651	-5.953	11.255	1.000
HD Severity: Low Vector Habitat<br>HD Frequency: 2 per 10-year period	-1.286	-9.890	7.318	1.000
HD Severity: Medium Vector Habitat<br>HD Frequency: 1 per 10-year period	2.126	-6.478	10.731	1.000
HD Severity: Medium Vector Habitat<br>HD Frequency: 2 per 10-year period	-4.943	-13.548	3.661	0.707
HD Severity: High Vector Habitat<br>HD Frequency: 1 per 10-year period	-0.335	-8.939	8.269	1.000
HD Severity: High Vector Habitat<br>HD Frequency: 2 per 10-year period	-4.148	-12.752	4.456	0.905
HD Severity: 70% Mortality				
HD Severity: Low Vector Habitat<br>HD Frequency: 1 per 10-year period	2.966	-5.639	11.570	0.998
HD Severity: Low Vector Habitat<br>HD Frequency: 2 per 10-year period	0.240	-8.364	8.845	1.000
HD Severity: Medium Vector Habitat<br>HD Frequency: 1 per 10-year period	0.635	-7.970	9.239	1.000
HD Severity: Medium Vector Habitat<br>HD Frequency: 2 per 10-year period	-9.530	-18.134	-0.925	**0.018**
HD Severity: High Vector Habitat<br>HD Frequency: 1 per 10-year period	-2.529	-11.133	6.075	1.000
HD Severity: High Vector Habitat<br>HD Frequency: 2 per 10-year period	-17.209	-25.814	-8.605	**<0.001**
**HD Timing: 2 months after CWD Introduction**				
HD Severity: 20% Mortality				
HD Severity: Low Vector Habitat<br>HD Frequency: 1 per 10-year period	-0.054	-8.659	8.550	1.000
HD Severity: Low Vector Habitat<br>HD Frequency: 2 per 10-year period	4.378	-4.227	12.982	0.857
HD Severity: Medium Vector Habitat<br>HD Frequency: 1 per 10-year period	3.283	-5.321	11.887	0.993
HD Severity: Medium Vector Habitat	-4.676	-13.280	3.928	0.782

HD Frequency: 2 per 10-year period				
HD Severity: High Vector Habitat<br>HD Frequency: 1 per 10-year period	-0.835	-9.440	7.769	1.000
HD Severity: High Vector Habitat<br>HD Frequency: 2 per 10-year period	-6.329	-14.934	2.275	0.332
HD Severity: 70% Mortality				
HD Severity: Low Vector Habitat<br>HD Frequency: 1 per 10-year period	-3.074	-11.678	5.531	0.997
HD Severity: Low Vector Habitat<br>HD Frequency: 2 per 10-year period	0.078	-8.527	8.682	1.000
HD Severity: Medium Vector Habitat<br>HD Frequency: 1 per 10-year period	-5.668	-14.273	2.936	0.497
HD Severity: Medium Vector Habitat<br>HD Frequency: 2 per 10-year period	-10.967	-19.571	-2.363	**0.003**
HD Severity: High Vector Habitat<br>HD Frequency: 1 per 10-year period	-1.812	-10.417	6.792	1.000
HD Severity: High Vector Habitat<br>HD Frequency: 2 per 10-year period	-14.369	-22.973	-5.765	**<0.001**
**HD Timing: 26 months after CWD Introduction**				
HD Severity: 20% Mortality				
HD Severity: Low Vector Habitat<br>HD Frequency: 1 per 10-year period	1.380	-7.225	9.984	1.000
HD Severity: Low Vector Habitat<br>HD Frequency: 2 per 10-year period	-4.674	-13.279	3.930	0.782
HD Severity: Medium Vector Habitat<br>HD Frequency: 1 per 10-year period	-1.425	-10.029	7.180	1.000
HD Severity: Medium Vector Habitat<br>HD Frequency: 2 per 10-year period	-4.284	-12.888	4.320	0.878
HD Severity: High Vector Habitat<br>HD Frequency: 1 per 10-year period	-3.448	-12.052	5.157	0.986
HD Severity: High Vector Habitat<br>HD Frequency: 2 per 10-year period	-6.576	-15.180	2.028	0.282
HD Severity: 70% Mortality				
HD Severity: Low Vector Habitat<br>HD Frequency: 1 per 10-year period	-4.373	-12.977	4.231	0.858
HD Severity: Low Vector Habitat<br>HD Frequency: 2 per 10-year period	-4.129	-12.734	4.475	0.908
HD Severity: Medium Vector Habitat<br>HD Frequency: 1 per 10-year period	-4.489	-13.094	4.115	0.830
HD Severity: Medium Vector Habitat<br>HD Frequency: 2 per 10-year period	-15.651	-24.255	-7.046	**<0.001**
HD Severity: High Vector Habitat	-13.921	-22.526	-5.317	**<0.001**

HD Frequency: 1 per 10-year period				
HD Severity: High Vector Habitat				
HD Frequency: 2 per 10-year period	-22.826	-31.431	-14.222	**<0.001**

Table S5: Results of Dunnett's post-hoc analysis for total CWD positive deer. Significant differences from HD-control are indicated in bold.

Source of Variation	Difference in Means	Lower CI	Upper CI	*P*
**HD Timing: 2 months before CWD Introduction**				
HD Severity: 20% Mortality				
HD Severity: Low Vector Habitat				
HD Frequency: 1 per 10-year period	10.247	-18.398	38.892	0.997
HD Severity: Low Vector Habitat				
HD Frequency: 2 per 10-year period	-4.645	-33.290	24.000	1.000
HD Severity: Medium Vector Habitat				
HD Frequency: 1 per 10-year period	9.801	-18.844	38.447	0.999
HD Severity: Medium Vector Habitat				
HD Frequency: 2 per 10-year period	-17.646	-46.291	10.999	0.602
HD Severity: High Vector Habitat				
HD Frequency: 1 per 10-year period	-1.814	-30.460	26.831	1.000
HD Severity: High Vector Habitat				
HD Frequency: 2 per 10-year period	-13.619	-42.264	15.026	0.915
HD Severity: 70% Mortality				
HD Severity: Low Vector Habitat				
HD Frequency: 1 per 10-year period	9.776	-18.869	38.421	0.999
HD Severity: Low Vector Habitat				
HD Frequency: 2 per 10-year period	0.555	-28.090	29.200	1.000
HD Severity: Medium Vector Habitat				
HD Frequency: 1 per 10-year period	2.754	-25.891	31.399	1.000
HD Severity: Medium Vector Habitat				
HD Frequency: 2 per 10-year period	-30.493	-59.138	-1.848	**0.028**
HD Severity: High Vector Habitat				
HD Frequency: 1 per 10-year period	-8.874	-37.519	19.771	1.000
HD Severity: High Vector Habitat				
HD Frequency: 2 per 10-year period	-54.122	-82.767	-25.477	**<0.001**
**HD Timing: 2 months after CWD Introduction**				
HD Severity: 20% Mortality				
HD Severity: Low Vector Habitat				
HD Frequency: 1 per 10-year period	0.046	-28.599	28.691	1.000
HD Severity: Low Vector Habitat				
HD Frequency: 2 per 10-year period	11.422	-17.223	40.068	0.987
HD Severity: Medium Vector Habitat	9.534	-19.111	38.179	0.999

HD Frequency: 1 per 10-year period				
HD Severity: Medium Vector Habitat				
HD Frequency: 2 per 10-year period	-15.335	-43.980	13.310	0.801
HD Severity: High Vector Habitat				
HD Frequency: 1 per 10-year period	-0.544	-29.189	28.101	1.000
HD Severity: High Vector Habitat				
HD Frequency: 2 per 10-year period	-22.580	-51.225	6.065	0.243
<u>HD Severity: 70% Mortality</u>				
HD Severity: Low Vector Habitat				
HD Frequency: 1 per 10-year period	-5.873	-34.518	22.772	1.000
HD Severity: Low Vector Habitat				
HD Frequency: 2 per 10-year period	-0.352	-28.997	28.293	1.000
HD Severity: Medium Vector Habitat				
HD Frequency: 1 per 10-year period	-17.912	-46.557	10.733	0.579
HD Severity: Medium Vector Habitat				
HD Frequency: 2 per 10-year period	-35.183	-63.828	-6.538	**0.005**
HD Severity: High Vector Habitat				
HD Frequency: 1 per 10-year period	-7.773	-36.418	20.872	1.000
HD Severity: High Vector Habitat				
HD Frequency: 2 per 10-year period	-43.857	-72.502	-15.212	**<0.001**
**HD Timing: 26 months after CWD Introduction**				
<u>HD Severity: 20% Mortality</u>				
HD Severity: Low Vector Habitat				
HD Frequency: 1 per 10-year period	6.689	-21.956	35.334	1.000
HD Severity: Low Vector Habitat				
HD Frequency: 2 per 10-year period	-14.667	-43.312	13.978	0.851
HD Severity: Medium Vector Habitat				
HD Frequency: 1 per 10-year period	-4.347	-32.992	24.298	1.000
HD Severity: Medium Vector Habitat				
HD Frequency: 2 per 10-year period	-13.238	-41.883	15.407	0.934
HD Severity: High Vector Habitat				
HD Frequency: 1 per 10-year period	-12.039	-40.684	16.606	0.975
HD Severity: High Vector Habitat				
HD Frequency: 2 per 10-year period	-23.459	-52.104	5.186	0.198
<u>HD Severity: 70% Mortality</u>				
HD Severity: Low Vector Habitat				
HD Frequency: 1 per 10-year period	-14.968	-43.613	13.677	0.829
HD Severity: Low Vector Habitat				
HD Frequency: 2 per 10-year period	-9.930	-38.575	18.715	0.998
HD Severity: Medium Vector Habitat				
HD Frequency: 1 per 10-year period	-12.179	-40.824	16.466	0.972
HD Severity: Medium Vector Habitat	-48.625	-77.270	-19.980	**<0.001**

HD Frequency: 2 per 10-year period				
HD Severity: High Vector Habitat<br>HD Frequency: 1 per 10-year period	-44.981	-73.626	-16.336	**<0.001**
HD Severity: High Vector Habitat<br>HD Frequency: 2 per 10-year period	-70.282	-98.927	-41.637	**<0.001**

Table S6: Results of Dunnett's post-hoc analysis for CWD prevalence.

Source of Variation	Difference in Means	Lower CI	Upper CI	*P*
**HD Timing: 2 months before CWD Introduction**				
HD Severity: 20% Mortality				
HD Severity: Low Vector Habitat<br>HD Frequency: 1 per 10-year period	0.001	-0.002	0.004	0.999
HD Severity: Low Vector Habitat<br>HD Frequency: 2 per 10-year period	0.000	-0.004	0.003	1.000
HD Severity: Medium Vector Habitat<br>HD Frequency: 1 per 10-year period	0.001	-0.002	0.004	0.992
HD Severity: Medium Vector Habitat<br>HD Frequency: 2 per 10-year period	-0.001	-0.004	0.002	0.999
HD Severity: High Vector Habitat<br>HD Frequency: 1 per 10-year period	0.000	-0.003	0.003	1.000
HD Severity: High Vector Habitat<br>HD Frequency: 2 per 10-year period	0.000	-0.003	0.003	1.000
HD Severity: 70% Mortality				
HD Severity: Low Vector Habitat<br>HD Frequency: 1 per 10-year period	0.001	-0.002	0.005	0.944
HD Severity: Low Vector Habitat<br>HD Frequency: 2 per 10-year period	0.001	-0.002	0.004	1.000
HD Severity: Medium Vector Habitat<br>HD Frequency: 1 per 10-year period	0.002	-0.001	0.005	0.629
HD Severity: Medium Vector Habitat<br>HD Frequency: 2 per 10-year period	0.000	-0.003	0.003	1.000
HD Severity: High Vector Habitat<br>HD Frequency: 1 per 10-year period	0.002	-0.001	0.005	0.451
HD Severity: High Vector Habitat<br>HD Frequency: 2 per 10-year period	0.001	-0.003	0.004	1.000
**HD Timing: 2 months after CWD Introduction**				
HD Severity: 20% Mortality				
HD Severity: Low Vector Habitat<br>HD Frequency: 1 per 10-year period	0.000	-0.003	0.003	1.000
HD Severity: Low Vector Habitat<br>HD Frequency: 2 per 10-year period	0.002	-0.002	0.005	0.894

HD Severity: Medium Vector Habitat HD Frequency: 1 per 10-year period	0.001	-0.002	0.004	0.995
HD Severity: Medium Vector Habitat HD Frequency: 2 per 10-year period	-0.001	-0.004	0.003	1.000
HD Severity: High Vector Habitat HD Frequency: 1 per 10-year period	0.000	-0.003	0.004	1.000
HD Severity: High Vector Habitat HD Frequency: 2 per 10-year period	-0.001	-0.005	0.002	0.970
HD Severity: 70% Mortality				
HD Severity: Low Vector Habitat HD Frequency: 1 per 10-year period	0.000	-0.003	0.003	1.000
HD Severity: Low Vector Habitat HD Frequency: 2 per 10-year period	0.001	-0.002	0.004	1.000
HD Severity: Medium Vector Habitat HD Frequency: 1 per 10-year period	0.000	-0.004	0.003	1.000
HD Severity: Medium Vector Habitat HD Frequency: 2 per 10-year period	0.000	-0.004	0.003	1.000
HD Severity: High Vector Habitat HD Frequency: 1 per 10-year period	0.002	-0.001	0.006	0.341
HD Severity: High Vector Habitat HD Frequency: 2 per 10-year period	0.002	-0.001	0.006	0.304
**HD Timing: 26 months after CWD Introduction**				
HD Severity: 20% Mortality				
HD Severity: Low Vector Habitat HD Frequency: 1 per 10-year period	0.001	-0.002	0.004	1.000
HD Severity: Low Vector Habitat HD Frequency: 2 per 10-year period	-0.001	-0.004	0.002	0.992
HD Severity: Medium Vector Habitat HD Frequency: 1 per 10-year period	0.000	-0.003	0.003	1.000
HD Severity: Medium Vector Habitat HD Frequency: 2 per 10-year period	0.000	-0.003	0.003	1.000
HD Severity: High Vector Habitat HD Frequency: 1 per 10-year period	-0.001	-0.004	0.003	1.000
HD Severity: High Vector Habitat HD Frequency: 2 per 10-year period	-0.001	-0.004	0.002	1.000
HD Severity: 70% Mortality				
HD Severity: Low Vector Habitat HD Frequency: 1 per 10-year period	-0.001	-0.004	0.002	0.998
HD Severity: Low Vector Habitat HD Frequency: 2 per 10-year period	0.000	-0.003	0.003	1.000
HD Severity: Medium Vector Habitat HD Frequency: 1 per 10-year period	0.001	-0.003	0.004	1.000

HD Severity: Medium Vector Habitat<br>HD Frequency: 2 per 10-year period	-0.002	-0.005	0.001	0.611
HD Severity: High Vector Habitat<br>HD Frequency: 1 per 10-year period	-0.001	-0.005	0.002	0.983
HD Severity: High Vector Habitat<br>HD Frequency: 2 per 10-year period	-0.001	-0.005	0.002	0.959

Table S7: Results of Dunnett's post-hoc analysis for deer population density. Significant differences from CWD-control are indicated in bold.

Treatment Group	Difference in Means	Lower CI	Upper CI	*P*
**CWD presence (No HD)**	-0.067	-2.009	1.875	1.000
**HD Timing: 2 months before CWD Introduction**				
HD Severity: 20% Mortality				
HD Severity: Low Vector Habitat<br>HD Frequency: 1 per 10-year period	-0.186	-2.128	1.756	1.000
HD Severity: Low Vector Habitat<br>HD Frequency: 2 per 10-year period	-0.401	-2.343	1.541	1.000
HD Severity: Medium Vector Habitat<br>HD Frequency: 1 per 10-year period	-0.374	-2.316	1.568	1.000
HD Severity: Medium Vector Habitat<br>HD Frequency: 2 per 10-year period	-1.043	-2.985	0.900	0.801
HD Severity: High Vector Habitat<br>HD Frequency: 1 per 10-year period	-0.517	-2.459	1.425	1.000
HD Severity: High Vector Habitat<br>HD Frequency: 2 per 10-year period	-1.310	-3.252	0.632	0.463
HD Severity: 70% Mortality				
HD Severity: Low Vector Habitat<br>HD Frequency: 1 per 10-year period	-0.589	-2.531	1.353	1.000
HD Severity: Low Vector Habitat<br>HD Frequency: 2 per 10-year period	-1.006	-2.948	0.936	0.843
HD Severity: Medium Vector Habitat<br>HD Frequency: 1 per 10-year period	-1.589	-3.531	0.353	0.201
HD Severity: Medium Vector Habitat<br>HD Frequency: 2 per 10-year period	-3.471	-5.413	-1.529	**<0.001**
HD Severity: High Vector Habitat<br>HD Frequency: 1 per 10-year period	-2.813	-4.755	-0.871	**<0.001**
HD Severity: High Vector Habitat<br>HD Frequency: 2 per 10-year period	-6.142	-8.084	-4.200	**<0.001**
**HD Timing: 2 months after CWD Introduction**				
HD Severity: 20% Mortality				
HD Severity: Low Vector Habitat	-0.204	-2.146	1.738	1.000

HD Frequency: 1 per 10-year period				
HD Severity: Low Vector Habitat HD Frequency: 2 per 10-year period	-0.402	-2.344	1.540	1.000
HD Severity: Medium Vector Habitat HD Frequency: 1 per 10-year period	-0.383	-2.325	1.559	1.000
HD Severity: Medium Vector Habitat HD Frequency: 2 per 10-year period	-1.042	-2.984	0.900	0.802
HD Severity: High Vector Habitat HD Frequency: 1 per 10-year period	-0.496	-2.438	1.446	1.000
HD Severity: High Vector Habitat HD Frequency: 2 per 10-year period	-1.303	-3.245	0.639	0.472
HD Severity: 70% Mortality				
HD Severity: Low Vector Habitat HD Frequency: 1 per 10-year period	-0.577	-2.519	1.365	1.000
HD Severity: Low Vector Habitat HD Frequency: 2 per 10-year period	-0.992	-2.934	0.950	0.857
HD Severity: Medium Vector Habitat HD Frequency: 1 per 10-year period	-1.555	-3.497	0.387	0.225
HD Severity: Medium Vector Habitat HD Frequency: 2 per 10-year period	-3.444	-5.386	-1.502	**<0.001**
HD Severity: High Vector Habitat HD Frequency: 1 per 10-year period	-2.812	-4.754	-0.870	**<0.001**
HD Severity: High Vector Habitat HD Frequency: 2 per 10-year period	-6.109	-8.051	-4.167	**<0.001**
**HD Timing: 26 months after CWD Introduction**				
HD Severity: 20% Mortality				
HD Severity: Low Vector Habitat HD Frequency: 1 per 10-year period	-0.272	-2.214	1.670	1.000
HD Severity: Low Vector Habitat HD Frequency: 2 per 10-year period	-0.443	-2.385	1.499	1.000
HD Severity: Medium Vector Habitat HD Frequency: 1 per 10-year period	-0.589	-2.531	1.353	1.000
HD Severity: Medium Vector Habitat HD Frequency: 2 per 10-year period	-1.317	-3.259	0.625	0.455
HD Severity: High Vector Habitat HD Frequency: 1 per 10-year period	-0.792	-2.734	1.150	0.983
HD Severity: High Vector Habitat HD Frequency: 2 per 10-year period	-1.840	-3.782	0.102	0.078
HD Severity: 70% Mortality				
HD Severity: Low Vector Habitat HD Frequency: 1 per 10-year period	-0.670	-2.612	1.272	0.999
HD Severity: Low Vector Habitat	-1.072	-3.014	0.870	0.766

HD Frequency: 2 per 10-year period				
HD Severity: Medium Vector Habitat<br>HD Frequency: 1 per 10-year period	-2.094	-4.036	-0.152	**0.024**
HD Severity: Medium Vector Habitat<br>HD Frequency: 2 per 10-year period	-3.694	-5.636	-1.752	**<0.001**
HD Severity: High Vector Habitat<br>HD Frequency: 1 per 10-year period	-3.792	-5.734	-1.850	**<0.001**
HD Severity: High Vector Habitat<br>HD Frequency: 2 per 10-year period	-6.495	-8.437	-4.553	**<0.001**
